# Bacteriophage application to control the contaminated water with *Shigella*

**DOI:** 10.1038/srep22636

**Published:** 2016-03-14

**Authors:** Jin Woo Jun, Sib Sankar Giri, Hyoun Joong Kim, Sae Kil Yun, Cheng Chi, Ji Young Chai, Byeong Chun Lee, Se Chang Park

**Affiliations:** 1 College of Veterinary Medicine and Research Institute for Veterinary Science, Seoul National University, Seoul, 151-742, Republic of Korea; 2 Departments of Rheumatology, Bundang Jesaeng Hospital, Seongnam, Republic of Korea

## Abstract

*Shigella* is one of the most important waterborne and foodborne pathogens around the world. Emergence of antibiotic-resistant *Shigella* has made the development of alternatives to conventional antibiotics necessary. In this study, a virulent *Myoviridae* bacteriophage, pSs-1 was isolated from environmental water in South Korea and showed infectivity to *S. flexneri* as well as *S. sonnei* strains. One-step growth analysis showed that pSs-1 has a short latent period (25 min) and a large burst size (97 PFU/cell). According to the genomic analysis, pSs-1 contains 164,999 bp of genome with a G + C content of 35.54% and it is considered as a member of the T4-like bacteriophage group. These results showed that pSs-1 may have potential as a biocontrol agent instead of conventional antibiotics for shigellosis.

*Shigella* is one of the most important waterborne and foodborne bacterial pathogens in the world^1^. It is usually related to the ingestion of contaminated water and food^1^. *Shigella* is human-adapted *Escherichia coli* and causes dysentery, spreading efficiently via low-dose fecal-oral transmission route^1^. It has been known that the majority shigellosis cases occur in developing countries and most of the patients are children under 5 years of age including infants^1^. However, numerous shigellosis cases are reported every year by military personnel and travelers in developed countries^2^.

The genus *Shigella* includes four species: *S. dysenteriae, S. flexneri, S. boydii*, and *S. sonnei*^3^. *S. flexneri* is the most commonly associated with shigellosis outbreaks in developing countries and causes approximately 2 estimated million cases per year worldwide^4^,^5^. *S. sonnei* has been the predominant agent responsible for dysentery in developed countries but is an emerging problem in developing areas^6^. The combination of increased incidence and excessive antimicrobial resistance among globally disseminated *Shigella* populations indicates that the development of effective control method will be increasingly important for long-term prevention of dysentery and associated morbidity and mortality^7^.

Bacteriophage (phage) can lyse a bacterial cell with acute specificity, which allows for the treatment of a targeted bacterial infection without the disruption of natural host microflora^8^. Phage therapy has been proposed for the treatment of human bacterial infections since phages were discovered in 1915 and 1917^9^. After a short period of phage therapy development, the developmental focus of antimicrobial therapy shifted from phage therapy to chemotherapy^9^. On the other hand, phage research has continued in Eastern Europe and the former Soviet Union, enabling phage therapy clinically^10^. However, more research on phage therapy has been recommended recently because of increasing risk of drug-resistant bacteria, the limited choice of effective treatments, and the declining development of novel antibiotics^9^. Our current study presented the isolation and characterization of a virulent *Myoviridae* phage, designated as pSs-1. The biological properties of pSs-1 were evaluated, and it showed its efficient bacteriolytic activity against *S. flexneri* as well as *S. sonnei*. Finally, its genome was completely sequenced and analyzed comparatively with its related phages. The main aim of this study was to examine the potential of phage as a biocontrol agent that can be used to control the contaminated water with *Shigella*.

## Results and Discussion

### Isolation and characterization of phage

In previous study, Jun and colleagues reported two virulent *Shigella* phages, a *Siphoviridae* phage pSf-1 infecting *S. flexneri*^11^ and a *Podoviridae* phage pSb-1 infecting *S. boydii*^12^. pSf-1 was able to infect all of the *S. flexneri* and most of *S. sonnei* strains, forming clear plaques^11^; pSb-1 was able to infect all of the *S. boydii* strains and formed clear plaques^12^. Although the virulent *Shigella* phages, pSf-1 and pSb-1 showed the potential usefulness against shigellosis as reported previously, the two phages were considered to present restricted effectiveness because *S. sonnei* ATCC^®^ 11060 was not infected by any of two phages^11^,^12^. This study aimed to isolate a virulent phage infecting *S. sonnei* strains in order to make the best combination of various phages for use in a phage cocktail as previously noted that the isolation of *S. sonnei* phage was needed^12^.

Previous research in our laboratory led to the isolation of *S. sonnei* phage as a first priority rather than the isolation of different phages infecting other *Shigella* species. A virulent *Shigella* phage, pSs-1 infecting *S. sonnei* and *S. flexneri* was isolated from the Hongjecheon stream in Seoul in April 2012. From the isolated phages, pSs-1 was selected for further studies depending on the clarity of plaque and the phage titer after a single propagation.

**Figure 1:**
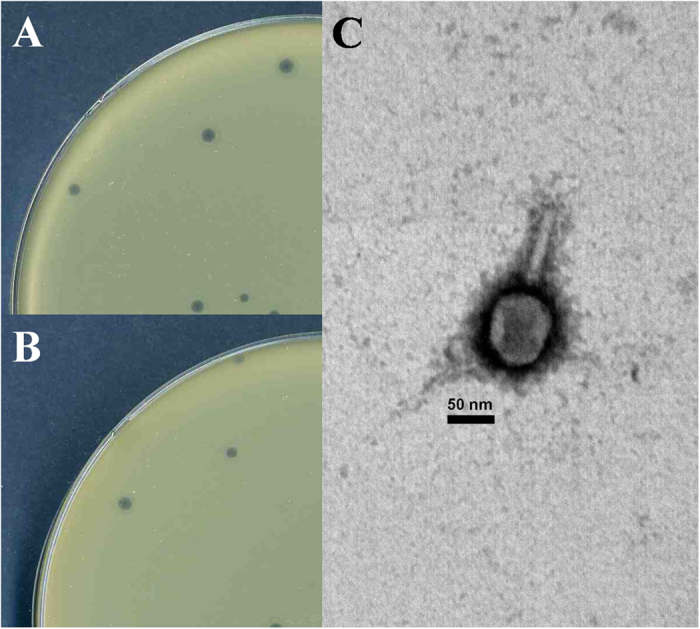
Phage plaques formed in double-layer agar plates and electron micrograph of negatively stained phage pSs-1. (**A**) phage plaques formed in agar plates with *S. sonnei* ATCC^®^ 25931, (**B**) phage plaques formed in agar plates with *S. flexneri* ATCC^®^ 29903, and (**C**) electron micrograph of pSs-1. The bar corresponds to 50 nm.

**Figure 2:**
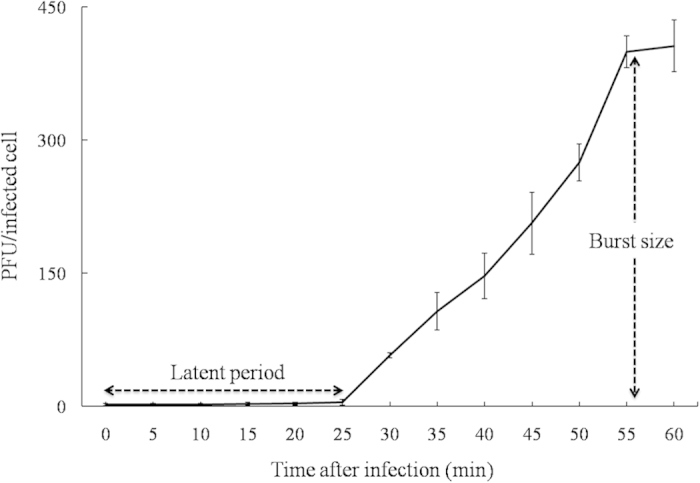
One-step growth curve of pSs-1. The error bars indicate standard deviations.

The morphology of pSs-1 places it in the family *Myoviridae* according to the classification system of Ackermann^13^ (Fig. 1C). The tail length and width were 120 ± 7 nm (mean ± SD) (*n* = 10) and 18 ± 2 nm (*n* = 10), respectively, and the head diameter was 98 ± 4 nm (*n* = 10). pSs-1 inhibited all of the *S. sonnei* and *S. flexneri* strains, producing clear plaques on all of the strains except one *S. flexneri* strain, ATCC^®^ 12022 (turbid plaque). However, no *S. boydii* strain was infected by pSs-1. The high EOP value was obtained with *S. flexneri* ATCC^®^ 29903 (Fig. 1B) although no strain had a higher value than the indicator host strain, *S. sonnei* ATCC^®^ 25931 (Table 1). Furthermore, pSs-1 was not able to infect *Escherichia coli* strains used in this study. The result of one-step growth analysis revealed that pSs-1 had a short latent period (25 min) and a large burst size (97 PFU/cell) (Fig. 2).

**Figure 3:**
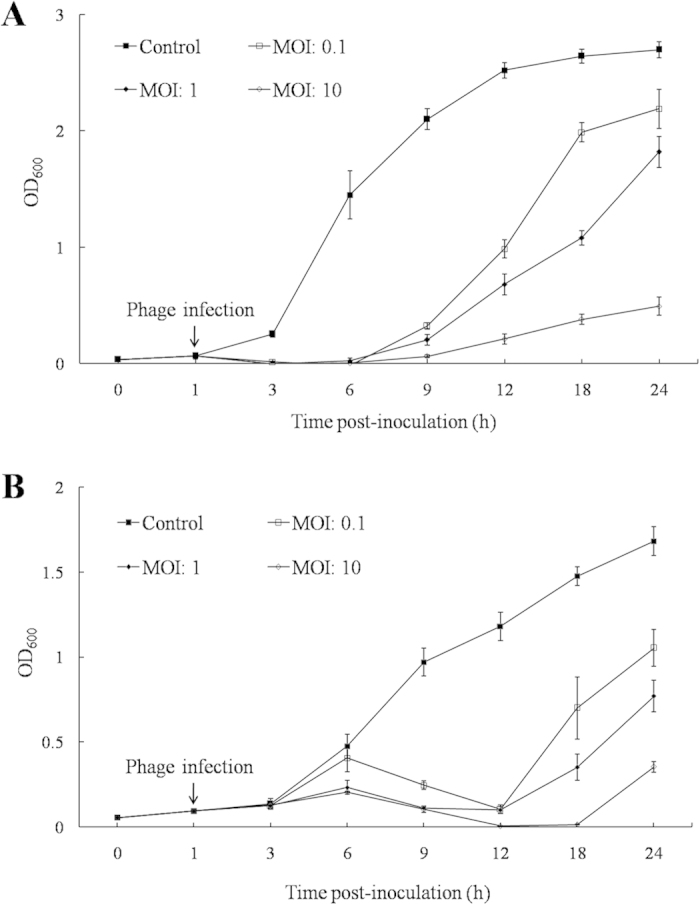
Time course of host cell lysis effect of pSs-1 against S. sonnei ATCC^®^ 25931 (**A**) and *S. flexneri* ATCC^®^ 29903 (**B**). Early exponential phase cultures of *S. sonnei* ATCC^®^ 25931 and *S. flexneri* ATCC^®^ 29903 were co-cultured with pSs-1 at MOIs of 0, 0.1, 1, and 10. The results are shown as mean + standard deviations from triplicate experiments.

The bacteriolytic effect of pSs-1 was tested on early phase cultures of *S. sonnei* ATCC^®^ 25931 (Fig. 3A) and *S. flexneri* ATCC^®^ 29903 (Fig. 3B). The OD_600_ values of the uninfected control culture (MOI: 0) continued to increase during the incubation period. In contrast, the growth of bacteria infected by pSs-1 was retarded at MOIs of 0.1, 1, and 10; the bacterial growth was inhibited most effectively at an MOI of 10. pSs-1 lysed *S. sonnei* (3 h after incubation) more readily than *S. flexneri* (12 h after incubation).

The effective bacteriolytic activity and high EOP value of pSs-1 against *S. sonnei* and *S. flexneri* strains indicated that pSs-1 could be used for control of both *S. sonnei* and *S. flexneri* although pSs-1 was proved to use only *S. sonnei* strain as a host bacterium; the propagation trials of pSs-1 using *S. flexneri* strains have not resulted in high titer for further study. These results conclude that the combination of pSf-1, pSb-1, and pSs-1 can inhibit all of the *Shigella* strains used in this study, increasing the possibility for *Shigella* control. The bacteriolytic activity of pSs-1 was found to be active for 1 h over the temperature range of 4–50 °C or the pH range of 5.0–9.0 toward *S. sonnei* and *S. flexneri* strains although significant reduction of its activity was observed at 50 °C (data not shown). This result emphasized that pSs-1 could be used in various natural environments, especially poor sanitation surroundings.

### Comparative genomic analysis of phage

According to the genome sequencing results, the genome size of pSs-1 is 164,999 bp, with a 35.54% total G + C content. The genomic sequence of the *Shigella* phage pSs-1 which was described in this study has been deposited in the GenBank database under the accession number KM501444. The predominant start codon was ATG; 10 ORFs such as ORF28, ORF51, ORF66, ORF67, ORF84, ORF112, ORF150, ORF178, ORF189, and ORF238 started with an uncommon start codon (GTG). ORFs with a length of at least 34 amino acids were selected. A total of 26 promoters, 14 transcriptional terminator regions, and 266 ORFs were predicted in the genome. However, only 121 ORFs (45.49%) were determined to be functional based on gene predictions and annotation of the genome. Concrete gene information such as positions, directions, sizes, molecular weights, and putative functions of each pSs-1 ORFs are shown in Supplementary Table S1. A total of 121 ORFs were determined to be functional. The functional analysis indicates that pSs-1 has similar functional system to those of T4-like phages. pSs-1 was proved to contain similar host lysis system to Shfl2 (virulent phage against *S. flexneri*) although *orf*217 showed higher similarity to SP18 (virulent phage against *S. sonnei*). Also, the predicted ORFs of phage structural genes were widely scattered across the entire genome although they were mostly located between *orf*155 and *orf*204 (74.46%, 35 ORFs in total).

**Figure 4:**
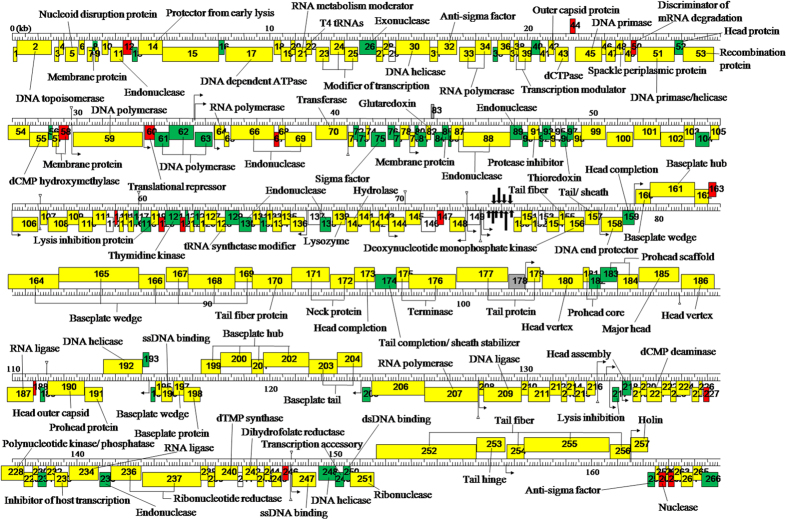
Functional genome map of phage pSs-1. Hypothetical functions of encoded proteins were determined by comparison of amino acid sequences to the non-redundant databank using BLASTP. The + and − stranded ORFs were colored as grey and white, respectively. The CoreGenes between pSs-1 and Shfl2, between pSs-1 and SP18, and between pSs-1 and T4 were colored as yellow, green, and red, respectively. The predicted tRNA was indicated with thick arrow. Putative promoters and terminators were indicated by a bent arrow and inverted triangle on a vertical line, respectively.

**Figure 5:**
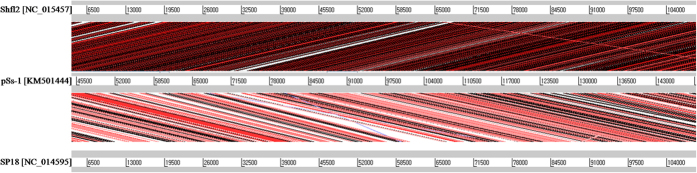
Genome comparison of pSs-1 to its related phages (Shfl2 and SP18) using Artemis Comparison Tool (ACT). Translated BLAST (tblastx, score cutoff: 40) was used to align translated genome sequences of phages. The blue and red lines represent the reverse and forward matches, respectively, and color intensity is proportional to the sequence homology. Nucleotide base-pairs were indicated between grey lines for each phage genome.

The comparative genome analysis of pSs-1 with T4-like phages, such as Shfl2 and SP18 revealed that pSs-1 had approximately 97% nucleotide sequence identity with Shfl2 and 186 homologous ORFs among its 266 ORFs (Fig. 4). In addition, pSs-1 showed approximately 93% nucleotide sequence identity with SP18 and 54 homologous ORFs (Fig. 4). According to the ACT comparison results, the pSs-1 genome revealed a higher degree of similarity to phage Shfl2 than phage SP18; the results showed the forward matches in the order of the genome of Shfl2 and SP18 (Fig. 5).

The high bacteriolytic activity of pSs-1 against both *S. sonnei* and *S. flexneri* may be attributed to the high nucleotide identity with Shfl2 and SP18^14^. All of the ORFs in pSs-1 exhibited homology to sequences of T4-like phages, such as Shfl2, SP18, T4, T6, AR1, RB14, RB32, RB51, and vB_EcoM_ACG-C40, reported in the GenBank database. T4-like phages, one of the best-characterized groups of phages present common characteristics: (i) morphology of the *Myoviridae* family; (ii) similar host range, the family *Enterobacteriacae*; (iii) large genome size in the range between 160 kbp and 250 kbp; (iv) similar G + C content, ranging from 35% to 43%^15^,^16^,^17^,^18^. Based on these results, pSs-1 is considered as a member of the T4-like phage group since it belongs to the *Myoviridae* family, infects *Shigella* species, contains relatively large genome (164,999 bp), and possesses G + C content of 35.54%. More than 200 T4-like phages have been examined and about 90% of T4-like phages grow on *Escherichia coli* or other *enterobacteria*, especially its close relatives such as *Klebsiella* and *Shigella*^17^,^18^.

A total of 10 tRNA genes (cove score 41.18–78.18) were identified^19^, which is more than the average number of tRNAs in T4-like phage group. The exceptionally large number of tRNAs (24 tRNAs) in Aeh1 may be attributed to its significantly large genome (233,234 bp) compared to those of the other T4-like phages^17^. Likewise, the genome of KVP40 (244,834 bp) encodes a large number of tRNAs^20^. Although the exact function of tRNA in phage is still not clear, it demonstrates that it may contribute to short latent period and large burst size of pSs-1 since tRNA in phage is known to influence its reproduction in the host, and facilitate the improvement of propagation and the reduction of latent period^21^. The genome of pSs-1 did not contain lysogeny genes and all of the ORFs had nothing in common with pathogenic factors.

In summary, it is considered as a universal problem as a lot of shigellosis cases are reported in developed countries as well as developing countries, although shigellosis had been regarded as a problem only in developing countries. In developing countries especially where poor hygiene standards occur, a safe year-round supply of drinking water remains a problem because the effective water treatment facility is often beyond their financial capacity^22^. The successful protection using phage against shigellosis was reported with animal experiments and the safety of phage administration through drinking water was reported with phage safety test in humans^8^,^23^,^24^,^25^. *Shigella* phage has potential uses to control or eradicate epidemic shigellosis in frequently affected area as phage is known to be cost-effective^26^. In developed countries where there are increasing concerns about antibiotic resistance, phages can be alternatives to conventional antibiotics. Phage research has been conducted continuously in Eastern Europe and Former Soviet Union countries, with leadership of the G. Eliava Institute of Bacteriophages, Microbiology and Virology in Tbilisi, Georgia. For many years, the Eliava Institute had developed and produced phages for the treatment and prophylaxis of bacterial infections including intestinal infections^27^. In a series of clinical trials, the therapeutic effectiveness of phages against infectious diseases was evaluated and no harmful effects were reported^27^. As the prevalence of a particular species of *Shigella* is generally considered to differ in various geographical areas^5^, a large-scale future screenings using *Shigella* environmental isolates from different geographical areas are required. This study may serve as a momentum to enhance the international collaborative research aiming control of waterborne infections, particularly shigellosis, on different continents.

## Methods

### Sampling, phage isolation and purification

Altogether 82 environmental water samples were collected from 5 different sampling sites, such as river and stream in South Korea between October 2011 and April 2012. Phage isolation was performed using a standard enrichment method using an indicator bacterium (*S. sonnei* ATCC^®^ 25931)^11^. All of the bacterial strains used in this study were cultured at 37 °C (Table 1). The conventional double-layer agar method was used for the examination of phage activity^28^. A single plaque was collected from the plate with a sterile Pasteur pipette and used to inoculate TSB containing 2 ml of log-phase *S. sonnei* ATCC^®^ 25931. This single plaque isolation procedure was repeated three successive times to obtain purified phages^11^. A *Shigella* phage, pSs-1, formed clear plaques in *S. sonnei* ATCC^®^ 25931 (Fig. 1A) and was selected for further studies.

### Phage morphology, host range and efficiency of plating (EOP)

In our study, every assay was performed in triplicate except electron microscopy. For the electron microscopy analysis, the phage suspension (7.8 × 10^8 ^PFU/ml) was concentrated and purified using continuous CsCl density gradient ultra-centrifugation^11^. The purified phages (1.0 × 10^11 ^PFU/ml, 6 ml of total volume) after the concentration/ purification using Polyethylene Glycol (PEG) and CsCl gradient centrifugation were negatively stained with 2% uranyl acetate. Electron micrographs were taken using a Zeiss TEM EM902 (Zeiss, Germany) at an accelerating voltage of 80 kV. The phage size was determined from at least 10 measurements^29^.

To evaluate the host range of pSs-1, its infectivity was tested on the bacterial strains used in this study. The presence of plaque formation and the number of plaques were determined after 24 h of incubation, and the EOP values were quantified by calculating the ratio of the PFU obtained with each phage-susceptible strain to the PFU obtained with the indicator strain.

### One-step growth and host cell lysis

Burst size and latent period of pSs-1 were determined by the one-step growth analysis as previously described^30^. Samples (100 μl) were collected at 5 min intervals and the titers were determined by the double-layer agar method. To evaluate the bacteriolytic activity of pSs-1 against *S. sonnei* ATCC^®^ 25931 and *S. flexneri* ATCC^®^ 29903, the absorbance (OD_600 nm_) was examined in order to determine the change of viable bacteria. After 1 h of the early-exponential phase bacterial incubation, the culture was divided into four 10 ml samples, which were co-cultured with phage suspensions at different multiplicities of infection (MOIs): 0, 0.1, 1, and 10. The preparations were incubated at 37 °C with shaking at 250 rpm. Bacteria not inoculated with pSs-1 (MOI: 0) were used as a control.

### Phage genome sequence analysis

The phage DNA was sequenced by GenoTech (Seoul, Korea) using Sanger sequencing and a Next Generation Sequencing System (NGS: Ion PGM 314 sequencer). The full length genome sequence was obtained by sequence assembly using CLC Genomics Workbench v.6.0.5. Contig gaps were filled by additional PCR and primer walking. Potential open reading frames (ORFs) that may encode gene products were predicted using GLIMMER and GeneMarkS, respectively^31^,^32^. The putative functions of the ORFs were analyzed by BLASTP searches at the National Center for Biotechnology Information (NCBI). Putative promoter regions were predicted using the Neural Network Promoter Prediction tool of the Berkeley Drosophila Genome Project (minimum promoter score: 0.9)^33^. Rho-independent transcription terminators were identified using Finding Terminators program (energy threshold value: −11)^34^. Transmembrane domains and signal sequence regions were predicted with the TMHMM program, ver. 2.0, and the SignalP 3.0 program, respectively^35^. The phage’s genome map was drawn using DNA Master^19^. The genome of pSs-1 was subjected to pairwise analysis using the Artemis Comparison Tool (ACT)^36^ with its close homologs, phage Shfl2 [GenBank accession number: NC_015457] and SP18 [GenBank accession number: NC_014595]. The protein sequence similarities of the phages were analyzed using CoreGenes3.0^37^.

## Additional Information

**Accession code**: The genomic sequence employed in the present study is available at 316 GenBank (No: KM501444).

**How to cite this article**: Jun, J. W. *et al*. Bacteriophage application to control the contaminated water with *Shigella. Sci. Rep.* **6**, 22636; doi: 10.1038/srep22636 (2016).

## Supplementary Material

Supplementary Information

## Figures and Tables

**Table 1:** Host range and EOPs of phage pSs-1 against all the bacterial strains used in this study.

**Bacterial species (*****n***)	**Strain**	**Infectivity**^a^	**EOPs**^b^
*Shigella sonnei* (4)	ATCC^®^ c 25931	++	(1.00)
	ATCC^®^ 29930	++	(0.77 ± 0.07)
	ATCC^®^ 11060	++	(0.73 ± 0.08)
	ATCC^®^ 9290	++	(0.65 ± 0.05)
*Shigella flexneri* (3)	ATCC^®^ 29903	++	(0.91 ± 0.05)
	ATCC^®^ 11836	++	(0.42 ± 0.03)
	ATCC^®^ 12022	+	(0.17 ± 0.07)
*Shigella boydii* (2)	ATCC^®^ 35966	−	−
	ATCC^®^ 8700	−	−
*Escherichia coli* (2)	ATCC^®^ 25922	−	−
	DH10B^d^	−	−

^a^++, clear plaque; +, turbid plaque; −, no plaque.

^b^The EOP (efficiency of plating) values were shown as the mean of observations at three different occasions.

^c^purchased from the American Type Culture Collection.

^d^purchased from Invitrogen.
