# Supplementary material for: Bacteriophage application to control the contaminated water with Shigella
Source: Sci Rep. 2016 Mar 14;6:22636. doi: 10.1038/srep22636 (PMC4789750; doi:10.1038/srep22636)
Supplement: Supplementary Information [file srep22636-s1.pdf]

Bacteriophage application to control the contaminated water with *Shigella*

Jin Woo Jun, Sib Sankar Giri, Hyoun Joong Kim, Sae Kil Yun, Cheng Chi, Ji Young Chai, Byeong Chun Lee, Se Chang Park

Supplementary Information

Table S1. Predicted genes and gene products of pSs-1.

Gene			Gene product			Amino acid Identity (%)	Putative function [organism] (E-value) {functional group [a]}	Predicted TMH and signal peptide	
ORF No.	Range	Strand	aa size	MW (kD)	pI			TMHHM	SignalP
1	40 - 189	-	50	4.9	9.50	98	hypothetical protein [Shigella phage Shfl2] (2e-21)	1	Y
2	186 - 1514	-	443	50.6	8.45	99	DNA topoisomerase [Shigella phage Shfl2] (0.0) {ii}	0	N
3	1652 - 1810	-	53	5.7	4.21	43	hypothetical protein [Shigella phage Shfl2] (0.021)	1	Y
4	1821 - 1976	-	52	5.6	7.82	75	hypothetical protein [Shigella phage Shfl2] (4e-17)	2	Y
5	2064 - 2519	-	152	17.0	9.90	99	nucleoid disruption protein [Shigella phage Shfl2] (3e-106) {v}	0	N
6	2579 - 2794	-	72	8.2	4.20	99	hypothetical protein [Shigella phage Shfl2] (6e-43)	0	N
7	2910 - 3107	-	66	7.5	9.40	95	hypothetical protein [Shigella phage Shfl2] (1e-36)	1	Y
8	3115 - 3228	-	38	4.3	9.52	61	membrane protein [Shigella phage SP18] (4e-06) {i}	1	N
9	3212 - 3325	-	38	4.6	7.84	94	hypothetical protein [Shigella phage Shfl2] (6e-14)	0	N
10	3472 - 3735	-	88	10.3	4.87	98	hypothetical protein [Shigella phage Shfl2] (2e-55)	0	N
11	3810 - 4286	-	159	18.2	7.66	97	endonuclease [Shigella phage Shfl2] (5e-112) {ii}	0	N
12	4300 - 4629	-	110	12.7	4.61	86	hypothetical protein [Enterobacteria phage T4] (6e-06)	0	N
13	4667 - 4861	-	65	7.6	6.20	48	hypothetical protein [Shigella phage SP18] (8e-13)	0	N
14	4890 - 5828	-	313	35.6	5.68	99	protector from early lysis [Shigella phage Shfl2] (0.0) {v}	0	N
15	5840 - 8017	-	726	82.8	5.84	97	hypothetical protein [Shigella phage Shfl2] (0.0)	0	N
16	8028 - 8231	-	68	8.2	6.58	78	hypothetical protein [Shigella phage SP18] (7e-24)	0	N
17	8286 - 10103	-	606	68.4	7.60	99	DNA dependent ATPase [Shigella phage Shfl2] (0.0) {ii}	0	N
18	10173 - 10433	-	87	9.4	7.71	99	hypothetical protein [Shigella phage Shfl2] (3e-55)	0	Y
19	10438 - 10809	-	124	14.3	4.94	96	hypothetical protein [Shigella phage Shfl2] (5e-78)	0	N
20	10812 - 10988	-	59	6.8	8.38	76	hypothetical protein [Shigella phage Shfl2] (6e-25)	0	N
21	10991 - 11410	-	140	16.6	4.76	99	RNA metabolism moderator [Shigella phage Shfl2] (1e-96) {iii}	0	N
22	11410 - 11625	-	72	8.5	5.00	97	T4 tRNAs [Shigella phage Shfl2] (9e-43) {iii}	0	N
23	11798 - 12286	-	163	16.6	4.76	93	modifier of transcription [Shigella phage Shfl2] (2e-102) {iii}	0	N
24	12363 - 12905	-	181	20.4	5.15	75	modifier of transcription [Shigella phage Shfl2] (1e-18) {iii}	0	N
25	12908 - 13408	-	167	20.0	5.97	87	modifier of transcription [Shigella phage Shfl2] (2e-101) {iii}	0	N
26	13472 - 14155	-	228	26.0	4.88	64	exonuclease [Shigella phage SP18] (4e-104) {ii}	0	N
27	14155 - 14397	-	81	9.3	5.36	98	hypothetical protein [Shigella phage Shfl2] (8e-48)	0	N
28	14390 - 14635	-	82	9.5	4.77	99	hypothetical protein [Shigella phage Shfl2] (5e-49)	0	N
29	14622 - 14882	-	87	9.9	7.85	94	hypothetical protein [Enterobacteria phage AR1] (2e-49)	0	N
30	14889 - 16208	-	440	50.0	7.61	99	DNA helicase [Shigella phage Shfl2] (0.0) {ii}	0	N
31	16205 - 16516	-	104	12.2	9.69	100	hypothetical protein [Shigella phage Shfl2] (1e-67)	0	N

32	16518 - 17264	-	249	29.1	9.95	98	anti-sigma factor [Shigella phage Shfl2] (3e-174) {iii}	0	N
33	17381 - 17983	-	201	23.4	5.86	99	RNA polymerase [Shigella phage Shfl2] (5e-145) {iii}	0	N
34	17980 - 18603	-	208	24.3	5.46	97	RNA polymerase [Shigella phage Shfl2] (5e-145) {iii}	0	N
35	18671 - 18853	-	61	7.1	4.27	41	hypothetical protein [Shigella phage SP18] (9e-07)	0	N
36	18862 - 19332	-	157	18.3	6.53	97	hypothetical protein [Shigella phage Shfl2] (9e-107)	0	N
37	19325 - 19477	-	51	5.8	5.73	98	hypothetical protein [Shigella phage Shfl2] (6e-26)	0	N
38	19486 - 19671	-	62	7.5	4.88	98	transcription modulator [Shigella phage Shfl2] (2e-34) {iii}	0	N
39	19664 - 20149	-	162	18.3	4.46	98	transcription modulator [Shigella phage Shfl2] (9e-110) {iii}	0	N
40	20158 - 20496	-	113	12.6	3.85	47	hypothetical protein [Shigella phage SP18] (5e-05)	0	N
41	20496 - 20708	-	71	8.4	5.52	60	hypothetical protein [Shigella phage Shfl2] (3e-22)	0	N
42	20806 - 21042	-	79	8.6	5.45	49	outer capsid protein [Shigella phage Shfl2] (2e-14) {i}	0	N
43	21085 - 21603	-	173	20.2	4.62	72	dCTPase [Shigella phage Shfl2] (2e-84) {iv}	0	N
44	21675 - 21875	+	67	7.2	10.00	95	hypothetical protein [Enterobacteria phage T4] (4e-36)	2	Y
45	21872 - 22900	-	343	39.9	9.31	99	DNA primase [Shigella phage Shfl2] (0.0) {ii}	0	N
46	22903 - 23067	-	55	6.0	5.39	98	hypothetical protein [Shigella phage Shfl2] (5e-28)	0	N
47	23069 - 23425	-	119	13.9	4.83	99	hypothetical protein [Shigella phage Shfl2] (8e-79)	0	N
48	23438 - 23731	-	98	11.1	4.62	97	spackle periplasmic protein [Shigella phage Shfl2] (5e-63) {v}	0	Y
49	23790 - 24047	-	86	10.2	9.88	95	hypothetical protein [Shigella phage Shfl2] (2e-45)	0	N
50	24049 - 24231	-	61	7.2	5.69	97	discriminator of mRNA degradation [Enterobacteria phage T4] (6e-33) {ii}	0	N
51	24290 - 25717	-	476	53.6	5.44	99	DNA primase/helicase [Shigella phage Shfl2] (0.0) {ii}	0	N
52	25727 - 26071	-	115	13.4	4.89	46	head vertex assembly protein [Shigella phage SP18] (5e-19) {i}	0	N
53	26064 - 27245	-	394	44.2	5.35	100	recombination protein [Shigella phage Shfl2] (0.0) {ii}	0	N
54	27323 - 28165	-	281	32.5	7.08	99	hypothetical protein [Shigella phage Shfl2] (0.0)	0	N
55	28162 - 28902	-	247	28.6	5.43	96	dCMP hydroxymethylase [Shigella phage Shfl2] (3e-176) {iv}	0	N
56	28910 - 29059	-	50	5.6	8.98	48	hypothetical protein [Shigella phage SP18] (1e-06)	1	Y
57	29056 - 29307	-	84	9.5	9.40	100	membrane protein [Shigella phage Shfl2] (4e-49) {i}	2	Y
58	29315 - 29695	-	127	14.3	7.55	98	membrane protein [Enterobacteria phage T4] (9e-85) {i}	0	Y
59	29876 - 32572	-	899	103.6	5.96	99	DNA polymerase [Shigella phage Shfl2] (0.0) {ii}	0	N
60	32651 - 33019	-	123	14.7	8.97	99	translational repressor protein [Enterobacteria phage T4] (6e-84) {ii}	0	N
61	33021 - 33584	-	188	21.5	7.66	54	DNA polymerase [Shigella phage SP18] (1e-63) {ii}	0	N
62	33586 - 34545	-	320	35.9	6.72	71	DNA polymerase [Shigella phage SP18] (2e-167) {ii}	0	N
63	34595 - 35281	-	229	25.0	4.89	61	DNA polymerase [Shigella phage SP18] (3e-95) {ii}	0	N
64	35337 - 35726	-	130	14.8	6.83	99	RNA polymerase [Shigella phage Shfl2] (9e-90) {iii}	0	N
65	35736 - 35924	-	63	7.5	5.55	97	hypothetical protein [Shigella phage Shfl2] (2e-36)	0	N
66	35980 - 37662	-	561	63.8	8.49	96	endonuclease [Shigella phage Shfl2] (0.0) {ii}	0	N
67	37659 - 37865	-	69	8.2	4.24	99	hypothetical protein [Enterobacteria phage T4] (2e-40)	0	N
68	37846 - 38109	-	88	10.3	4.21	99	hypothetical protein [Shigella phage Shfl2] (3e-55)	0	N

69	38106 - 39125	-	340	39.2	4.90	99	endonuclease [Shigella phage Shfl2] (0.0) {ii}	0	N
70	39302 - 40504	-	401	46.9	6.05	99	alpha-glucosyl-transferase [Shigella phage Shfl2] (0.0) {iv}	0	N
71	40571 - 40744	-	58	6.7	9.56	89	hypothetical protein [Shigella phage Shfl2] (4e-29)	0	N
72	40748 - 40951	-	68	8.0	9.42	43	hypothetical protein [Shigella phage SP18] (2e-08)	0	N
73	40920 - 41237	-	106	12.5	8.78	42	hypothetical protein [Shigella phage SP18] (3e-17)	0	N
74	41239 - 41382	-	48	5.8	4.14	100	hypothetical protein [Shigella phage Shfl2] (7e-25)	0	N
75	41441 - 41998	-	186	21.6	5.44	79	sigma factor [Shigella phage SP18] (5e-101) {iii}	0	N
76	42077 - 42346	-	90	10.8	5.64	52	hypothetical protein [Shigella phage SP18] (6e-26)	0	N
77	42343 - 42558	-	72	8.2	3.69	58	hypothetical protein [Shigella phage SP18] (2e-17)	0	N
78	42561 - 42887	-	109	12.8	9.70	98	hypothetical protein [Shigella phage Shfl2] (2e-70)	0	N
79	42940 - 43140	-	67	7.8	7.89	94	hypothetical protein [Shigella phage Shfl2] (5e-39)	0	N
80	43141 - 43272	-	44	5.2	9.60	93	hypothetical protein [Shigella phage SP18] (2e-20)	0	N
81	43280 - 43573	-	98	11.9	9.79	74	hypothetical protein [Shigella phage SP18] (1e-45)	0	N
82	43566 - 43748	-	61	7.1	9.37	66	hypothetical protein [Shigella phage Shfl2] (3e-18)	1	N
83	43773 - 43880	+	36	4.2	9.99	96	hypothetical protein [Enterobacteria phage RB14] (5e-09)	0	N
84	43906 - 44214	-	103	11.8	9.14	68	glutaredoxin [Shigella phage SP18] (4e-42) {iv}	0	N
85	44217 - 44429	-	71	8.0	9.22	38	membrane protein [Shigella phage SP18] (2e-07) {i}	1	N
86	44439 - 44552	-	38	4.5	4.54	92	hypothetical protein [Shigella phage SP18] (5e-16)	0	N
87	44545 - 45015	-	157	18.2	5.90	98	ribonucleotide reductase [Shigella phage Shfl2] (6e-110) {iv}	0	N
88	45012 - 46829	-	606	68.1	6.59	99	ribonucleotide reductase [Shigella phage Shfl2] (0.0) {iv}	0	N
89	46826 - 47299	-	158	18.2	8.96	83	endonuclease [Shigella phage SP18] (3e-90) {ii}	0	N
90	47342 - 47518	-	59	7.0	10.15	78	hypothetical protein [Shigella phage SP18] (3e-25)	0	N
91	47518 - 47964	-	149	17.0	4.37	92	host protease inhibitor [Shigella phage Shfl2] (9e-93) {v}	0	N
92	47948 - 48103	-	52	6.2	3.94	73	hypothetical protein [Shigella phage SP18] (2e-14)	0	N
93	48088 - 48408	-	107	12.6	4.48	74	hypothetical protein [Shigella phage SP18] (3e-52)	0	N
94	48419 - 48589	-	57	6.7	4.15	93	hypothetical protein [Shigella phage Shfl2] (9e-28)	0	N
95	48592 - 48804	-	71	8.0	7.79	100	hypothetical protein [Enterobacteria phage RB51] (4e-42)	0	N
96	48804 - 49067	-	88	10.1	6.71	87	thioredoxin [Shigella phage SP18] (2e-51) {iv}	0	N
97	49069 - 49311	-	81	9.5	7.91	79	hypothetical protein [Shigella phage SP18] (6e-36)	0	N
98	49298 - 49615	-	106	12.2	6.51	92	hypothetical protein [Shigella phage Shfl2] (6e-67)	0	N
99	49612 - 50541	-	310	36.0	9.45	97	hypothetical protein [Shigella phage Shfl2] (0.0)	0	N
100	50594 - 51595	-	334	39.1	5.91	92	hypothetical protein [Shigella phage Shfl2] (0.0)	0	N
101	51653 - 52675	-	341	39.6	9.12	92	hypothetical protein [Shigella phage Shfl2] (0.0)	0	N
102	52684 - 53574	-	297	34.1	9.17	97	hypothetical protein [Shigella phage Shfl2] (0.0)	0	N
103	53582 - 53983	-	134	15.4	6.54	92	hypothetical protein [Shigella phage Shfl2] (3e-62)	2	Y
104	54039 - 54566	-	176	20.8	6.65	68	hypothetical protein [Shigella phage SP18] (6e-82)	0	N
105	54627 - 54929	-	101	12.1	9.68	99	hypothetical protein [Shigella phage Shfl2] (9e-67)	0	N

106	55031 - 55999	-	323	36.5	5.01	99	hypothetical protein [Shigella phage Shfl2] (0.0)	0	N
107	56114 - 56359	-	82	9.6	4.69	98	hypothetical protein [Shigella phage Shfl2] (2e-48)	0	N
108	56389 - 57123	-	245	28.4	9.13	95	hypothetical protein [Shigella phage Shfl2] (3e-166)	0	N
109	57123 - 57584	-	154	18.0	9.33	91	hypothetical protein [Shigella phage Shfl2] (6e-79)	2	N
110	57593 - 58114	-	174	19.3	4.89	96	hypothetical protein [Shigella phage Shfl2] (5e-117)	0	N
111	58121 - 58654	-	178	20.8	5.20	84	hypothetical protein [Shigella phage Shfl2] (3e-106)	0	N
112	58656 - 58922	-	89	10.3	6.71	95	hypothetical protein [Enterobacteria phage RB14] (4e-55)	0	N
113	58983 - 59087	-	35	4.3	8.14	91	hypothetical protein [Enterobacteria phage T4] (4e-12)	1	N
114	59148 - 59321	-	58	6.8	5.27	100	hypothetical protein [Shigella phage Shfl2] (2e-32)	0	N
115	59311 - 59505	-	65	7.7	5.04	63	hypothetical protein [Shigella phage Shfl2] (1e-14)	0	N
116	59508 - 59711	-	68	7.7	4.59	93	hypothetical protein [Shigella phage Shfl2] (2e-34)	0	N
117	59711 - 59899	-	63	7.2	4.11	51	hypothetical protein [Shigella phage SP18] (7e-09)	0	N
118	59995 - 60381	-	129	14.7	5.53	56	hypothetical protein [Shigella phage SP18] (2e-45)	0	N
119	60378 - 60671	-	98	11.2	4.90	98	lysis inhibition protein [Shigella phage Shfl2] (8e-64) {v}	0	Y
120	60684 - 60896	-	71	8.4	10.19	100	hypothetical protein [Enterobacteria phage T4] (4e-42)	0	N
121	60939 - 61520	-	194	21.7	6.22	71	thymidine kinase [Shigella phage SP18] (2e-95) {iv}	0	N
122	61522 - 61710	-	63	7.3	4.12	100	hypothetical protein [Enterobacteria phage T4] (8e-34)	0	N
123	61707 - 61838	-	44	5.2	3.91	42	hypothetical protein [Shigella phage SP18] (0.003)	0	N
124	61889 - 62065	-	59	6.6	6.53	59	hypothetical protein [Shigella phage Shfl2] (5e-11)	0	N
125	62062 - 62274	-	71	8.2	5.51	71	hypothetical protein [Shigella phage SP18] (6e-28)	0	N
126	62265 - 62477	-	71	8.6	8.83	99	hypothetical protein [Shigella phage Shfl2] (7e-44)	0	N
127	62449 - 62928	-	160	17.6	5.03	97	hypothetical protein [Shigella phage Shfl2] (3e-110)	0	N
128	62925 - 63272	-	116	13.1	8.95	99	tRNA synthetase modifier [Shigella phage Shfl2] (4e-76) {iv}	0	Y
129	63265 - 63810	-	182	20.8	9.76	68	hypothetical protein [Shigella phage SP18] (1e-76)	0	Y
130	63818 - 64279	-	154	18.0	8.93	60	endonuclease [Shigella phage SP18] (2e-52) {ii}	0	N
131	64339 - 64617	-	93	11.0	5.45	99	hypothetical protein [Shigella phage Shfl2] (1e-58)	0	N
132	64617 - 64883	-	89	10.3	4.66	64	hypothetical protein [Shigella phage SP18] (5e-28)	0	N
133	64876 - 65097	-	74	8.3	4.07	95	hypothetical protein [Shigella phage Shfl2] (1e-42)	0	N
134	65097 - 65459	-	121	13.9	5.94	96	hypothetical protein [Shigella phage Shfl2] (5e-78)	0	N
135	65466 - 65795	-	110	12.9	8.89	96	hypothetical protein [Shigella phage Shfl2] (2e-71)	0	N
136	65792 - 66331	-	180	20.4	8.72	94	hypothetical protein [Shigella phage Shfl2] (2e-112)	0	N
137	66485 - 66958	-	158	17.8	8.91	99	hypothetical protein [Enterobacteria phage RB51] (6e-109)	0	N
138	66968 - 67384	-	139	16.4	9.28	72	endonuclease [Shigella phage SP18] (1e-68) {ii}	0	N
139	67444 - 67938	-	165	18.7	9.59	98	lysozyme [Shigella phage Shfl2] (6e-114) {v}	0	N
140	67975 - 68415	-	147	17.0	5.05	96	hydrolase [Shigella phage Shfl2] (9e-101) {v}	0	N
141	68412 - 68900	-	163	19.2	9.06	90	hypothetical protein [Shigella phage Shfl2] (1e-101)	2	N
142	68897 - 69271	-	125	14.7	8.81	86	hypothetical protein [Shigella phage Shfl2] (3e-61)	2	Y

143	69253 - 69645	-	131	15.1	9.67	85	hypothetical protein [Shigella phage Shfl2] (1e-64)	2	N
144	69614 - 70228	-	205	24.2	5.37	96	hypothetical protein [Shigella phage Shfl2] (5e-139)	0	N
145	70270 - 70863	-	198	22.2	6.06	99	hypothetical protein [Shigella phage Shfl2] (3e-133)	0	N
146	70906 - 71436	-	177	19.8	6.90	92	hypothetical protein [Enterobacteria phage RB32] (1e-111)	0	N
147	71493 - 71756	-	88	10.2	4.46	99	hypothetical protein [Enterobacteria phage T4] (2e-56)	0	N
148	71997 - 72560	-	188	20.9	9.43	97	hypothetical protein [Shigella phage Shfl2] (5e-124)	0	N
149	72686 - 73159	-	158	17.3	9.46	99	hypothetical protein [Enterobacteria phage AR1] (9e-107)	0	N
150	74479 - 74766	-	96	11.3	4.91	96	hypothetical protein [Shigella phage Shfl2] (2e-59)	0	N
151	74769 - 75179	-	137	16.2	4.85	98	hypothetical protein [Shigella phage Shfl2] (2e-93)	0	N
152	75181 - 75366	-	62	6.7	7.80	95	hypothetical protein [Shigella phage Shfl2] (2e-31)	2	Y
153	75437 - 75694	-	86	9.4	9.34	96	hypothetical protein [Enterobacteria phage RB51] (2e-50)	0	N
154	75767 - 76222	-	152	17.2	5.14	99	hypothetical protein [Shigella phage Shfl2] (8e-106)	0	N
155	76222 - 76464	-	81	8.8	4.26	96	tail fiber assembly protein [Shigella phage Shfl2] (1e-42) {i}	0	N
156	76464 - 77195	-	244	27.7	5.06	96	deoxynucleotide monophosphate kinase [Shigella phage Shfl2] (2e-169) {iv}	0	N
157	77245 - 77775	-	177	19.8	4.39	99	tail completion and sheath stabilizer protein [Shigella phage Shfl2] (1e-124) {i}	0	N
158	77882 - 78706	-	275	31.7	10.12	99	DNA end protector protein [Shigella phage Shfl2] (0.0) {ii}	0	N
159	78706 - 79158	-	151	17.7	9.75	66	head completion protein [Shigella phage SP18] (7e-69) {i}	0	N
160	79206 - 79796	+	197	23.0	5.92	99	baseplate wedge [Shigella phage Shfl2] (3e-140) {i}	0	N
161	79780 - 81507	+	576	63.2	5.28	99	baseplate hub protein [Shigella phage Shfl2] (0.0) {i}	0	N
162	81500 - 82036	+	179	20.2	4.46	97	hypothetical protein [Shigella phage Shfl2] (2e-121)	0	N
163	82037 - 82330	+	98	10.3	8.60	100	hypothetical protein [Enterobacteria phage T4] (1e-63)	0	N
164	82339 - 84321	+	661	74.4	4.62	99	baseplate wedge [Shigella phage Shfl2] (0.0) {i}	0	N
165	84318 - 87416	+	1033	119.2	5.11	99	baseplate wedge [Shigella phage Shfl2] (0.0) {i}	1	N
166	87409 - 88413	+	335	38.1	4.61	97	baseplate wedge [Shigella phage Shfl2] (0.0) {i}	0	N
167	88477 - 89343	+	289	31.0	5.09	99	baseplate wedge [Shigella phage Shfl2] (0.0) {i}	0	N
168	89343 - 91148	+	602	66.4	4.50	85	baseplate wedge [Shigella phage Shfl2] (0.0) {i}	0	N
169	91148 - 91807	+	220	24.1	5.18	68	baseplate wedge [Shigella phage Shfl2] (4e-104) {i}	0	N
170	91804 - 93354	+	517	55.5	5.85	65	tail fiber protein [Shigella phage Shfl2] (0.0) {i}	0	N
171	93364 - 94821	+	486	52.5	4.77	82	neck whisker protein [Shigella phage Shfl2] (0.0) {i}	0	N
172	94854 - 95783	+	310	34.7	4.93	99	neck protein [Shigella phage Shfl2] (0.0) {i}	0	N
173	95785 - 96555	+	257	29.6	4.57	99	head completion protein [Shigella phage Shfl2] (0.0) {i}	0	N
174	96597 - 97415	+	273	31.6	4.95	55	tail completion and sheath stabilizer protein [Shigella phage SP18] (9e-95) {i}	0	N
175	97424 - 97918	+	165	18.5	4.55	99	terminase [Shigella phage Shfl2] (2e-115) {ii}	0	N
176	97902 - 99734	+	611	69.8	5.53	100	terminase [Shigella phage Shfl2] (0.0) {ii}	0	N
177	99766 - 101745	+	660	71.4	4.83	99	tail sheath protein [Shigella phage Shfl2] (0.0) {i}	0	N
178	101785-102402	+	206	23.9	9.53	51	hypothetical protein [Enterobacteria phage T6] (3e-52)	0	N
179	102510-103001	+	164	18.5	4.67	100	tail tube protein [Shigella phage Shfl2] (3e-116) {i}	0	N

180	103085-104659	+	525	61.1	5.36	99	head vertex protein [Shigella phage Shfl2] (0.0) {i}	0	N
181	104659-104916	+	86	9.8	3.81	96	prohead core protein [Shigella phage Shfl2] (1e-21) {i}	0	N
182	104916-105341	+	142	16.0	9.98	67	prohead core protein [Shigella phage SP18] (1e-48) {i}	0	N
183	105341-105979	+	213	23.3	4.98	88	prohead core scaffold protein [Shigella phage SP18] (3e-131) {i}	0	N
184	106010-106819	+	270	29.9	4.58	99	prohead core scaffold protein [Shigella phage Shfl2] (0.0) {i}	0	N
185	106838-108403	+	522	56.2	5.34	99	major head protein [Shigella phage Shfl2] (0.0) {i}	0	N
186	108487-109770	+	428	47.1	4.70	99	head vertex protein [Shigella phage Shfl2] (0.0) {i}	0	N
187	109800-110804	-	335	37.7	5.65	98	RNA ligase [Shigella phage Shfl2] (0.0) {ii}	0	N
188	110814-110915	-	34	4.0	4.29	97	hypothetical protein [Enterobacteria phage T4] (2e-14)	0	N
189	111078-111263	-	62	7.2	10.35	59	hypothetical protein [Shigella phage SP18] (1e-04)	0	N
190	111383-112801	-	473	50.9	4.44	78	head outer capsid protein [Shigella phage Shfl2] (0.0) {i}	0	N
191	112811-113491	-	227	25.6	4.42	98	prohead protein [Shigella phage Shfl2] (6e-159) {i}	0	N
192	113542-115053	+	504	58.0	9.29	99	DNA helicase [Shigella phage Shfl2] (0.0) {ii}	0	N
193	115079-115309	+	77	8.9	4.24	75	hypothetical protein [Shigella phage SP18] (8e-23)	0	N
194	115365-115532	-	56	6.1	4.44	82	hypothetical protein [Shigella phage SP18] (4e-26)	0	N
195	115561-115785	-	75	9.1	4.85	95	hypothetical protein [Shigella phage Shfl2] (1e-44)	0	N
196	115785-116198	-	138	15.9	7.76	99	ssDNA binding protein [Shigella phage Shfl2] (7e-92) {iii}	0	N
197	116265-116663	-	133	15.2	4.57	100	baseplate wedge protein [Shigella phage Shfl2] (2e-89) {i}	0	N
198	116663-117289	-	209	24.0	5.57	99	baseplate protein [Shigella phage Shfl2] (4e-148) {i}	0	N
199	117340-118089	+	250	29.4	5.82	98	baseplate hub protein [Shigella phage Shfl2] (1e-178) {i}	0	N
200	118089-119264	+	392	44.5	5.21	99	baseplate hub protein [Shigella phage Shfl2] (0.0) {i}	0	N
201	119284-119742	+	153	17.3	5.01	99	baseplate hub protein [Shigella phage Shfl2] (5e-105) {i}	0	N
202	119739-121511	+	591	64.5	5.07	96	baseplate hub protein [Shigella phage Shfl2] (0.0) {i}	1	N
203	121520-122614	+	365	39.8	8.40	95	baseplate tail protein [Shigella phage Shfl2] (0.0) {i}	0	N
204	122614-123579	+	322	35.0	5.08	99	baseplate tail protein [Shigella phage Shfl2] (0.0) {i}	0	N
205	123608-123898	-	97	10.8	4.67	59	hypothetical protein [Shigella phage SP18] (1e-29)	0	N
206	123959-126016	-	686	76.0	6.05	99	hypothetical protein [Shigella phage Shfl2] (0.0)	0	N
207	126020-128074	-	685	76.1	5.73	72	RNA polymerase [Shigella phage Shfl2] (0.0) {iii}	0	N
208	128127-128315	-	63	7.2	4.50	90	hypothetical protein [Shigella phage Shfl2] (3e-34)	0	N
209	128312-129772	-	487	55.2	5.85	98	DNA ligase [Shigella phage Shfl2] (0.0) {ii}	0	N
210	129769-130038	-	90	10.9	7.82	98	hypothetical protein [Shigella phage Shfl2] (2e-58)	0	N
211	130038-130874	-	279	32.5	5.84	90	hypothetical protein [Shigella phage Shfl2] (0.0)	0	N
212	130871-131329	-	153	17.2	9.20	97	hypothetical protein [Shigella phage Shfl2] (2e-83)	0	N
213	131322-131537	-	72	8.3	4.94	49	hypothetical protein [Shigella phage Shfl2] (2e-14)	0	N
214	131542-131829	-	96	10.8	7.80	97	hypothetical protein [Shigella phage Shfl2] (1e-61)	0	N
215	131870-132235	-	122	14.2	6.05	100	hypothetical protein [Shigella phage Shfl2] (3e-85)	0	N
216	132303-132635	-	111	13.0	6.11	97	hypothetical protein [Shigella phage Shfl2] (2e-71)	0	N

217	133318-133566	-	83	9.4	8.06	89	lysis inhibition protein [Shigella phage SP18] (4e-42) {v}	0	N
218	133714-134049	-	112	12.2	5.30	80	head assembly protein [Shigella phage SP18] (3e-55) {i}	0	N
219	134106-134414	-	103	11.5	9.35	99	hypothetical protein [Shigella phage Shfl2] (1e-65)	0	N
220	134415-134651	-	79	9.5	9.71	95	hypothetical protein [Shigella phage Shfl2] (2e-46)	0	N
221	134651-135232	-	194	21.3	8.04	99	dCMP deaminase [Shigella phage Shfl2] (2e-139) {iv}	0	N
222	135229-135567	-	113	12.9	7.87	100	hypothetical protein [Shigella phage Shfl2] (5e-75)	0	N
223	135564-135800	-	79	8.8	5.75	99	hypothetical protein [Shigella phage Shfl2] (2e-46)	0	N
224	135794-136324	-	177	20.5	4.99	94	hypothetical protein [Shigella phage Shfl2] (6e-103)	0	N
225	136387-136662	-	92	10.2	4.87	99	hypothetical protein [Shigella phage Shfl2] (8e-58)	0	N
226	136665-136796	-	44	4.9	3.35	81	hypothetical protein [Shigella phage Shfl2] (6e-15)	0	N
227	136860-137057	-	66	7.7	6.71	95	hypothetical protein [Enterobacteria phage T4] (6e-38)	0	N
228	137057-137965	-	303	34.9	8.64	94	polynucleotide kinase/phosphatase [Shigella phage Shfl2] (0.0) {iv}	0	Y
229	137962-138282	-	107	12.2	8.55	98	hypothetical protein [Shigella phage Shfl2] (2e-69)	0	N
230	138286-138510	-	75	8.8	6.82	100	hypothetical protein [Shigella phage Shfl2] (6e-47)	0	N
231	138507-138806	-	100	11.7	8.76	65	hypothetical protein [Shigella phage SP18] (8e-37)	0	N
232	138803-139156	-	118	13.2	8.89	99	hypothetical protein [Shigella phage Shfl2] (8e-75)	1	N
233	139147-139653	-	169	19.2	6.42	100	inhibitor of host transcription [Shigella phage Shfl2] (6e-120) {iii}	0	N
234	139715-140839	-	375	43.6	5.02	99	RNA ligase [Shigella phage Shfl2] (0.0) {ii}	0	N
235	140892-141302	-	137	15.9	9.21	74	endonuclease [Shigella phage SP18] (2e-67) {ii}	0	N
236	141330-142508	-	393	46.1	4.93	92	ribonucleotide reductase [Shigella phage Shfl2] (0.0) {iv}	0	N
237	142560-144824	-	755	86.1	5.87	99	ribonucleotide reductase [Shigella phage Shfl2] (0.0) {iv}	0	N
238	144815-145102	-	96	11.0	8.98	99	hypothetical protein [Shigella phage Shfl2] (3e-63)	0	N
239	145095-145358	-	88	10.1	5.73	99	hypothetical protein [Shigella phage Shfl2] (3e-55)	0	N
240	145382-146242	-	287	33.2	6.07	81	dTMP synthase [Shigella phage Shfl2] (3e-177) {iv}	0	N
241	146242-146448	-	69	8.0	10.26	98	hypothetical protein [Enterobacteria phage vB_EcoM_ACG-C40] (9e-21)	0	N
242	146445-147029	-	195	21.8	6.09	96	dihydrofolate reductase [Shigella phage Shfl2] (3e-136) {iv}	0	N
243	147029-147274	-	82	9.8	4.05	99	hypothetical protein [Shigella phage Shfl2] (6e-50)	0	N
244	147285-147527	-	81	9.6	4.93	94	hypothetical protein [Shigella phage Shfl2] (4e-47)	0	N
245	147582-147962	-	127	15.0	5.62	92	hypothetical protein [Shigella phage Shfl2] (2e-78)	0	N
246	148008-148235	-	76	8.9	3.88	100	hypothetical protein [Enterobacteria phage T4] (3e-46)	0	N
247	148381-149289	-	303	33.6	4.82	99	ssDNA binding protein [Shigella phage Shfl2] (0.0) {iii}	0	N
248	149389-150042	-	218	26.1	9.37	75	DNA helicase [Shigella phage SP18] (1e-116) {ii}	0	N
249	150039-150377	-	113	12.9	4.47	64	transcription accessory protein [Shigella phage SP18] (4e-28) {iii}	0	N
250	150355-150624	-	90	10.5	5.04	60	dsDNA binding protein [Shigella phage SP18] (6e-23) {iii}	0	N
251	150633-151550	-	306	35.6	8.61	99	ribonuclease [Shigella phage Shfl2] (0.0) {ii}	0	N
252	151655-155527	+	1291	140.4	5.19	95	tail fiber protein [Shigella phage Shfl2] (0.0) {i}	0	N
253	155536-156651	+	372	40.3	5.16	96	tail hinge connector [Shigella phage Shfl2] (0.0) {i}	0	N

254	156714-157370	+	219	23.4	6.07	81	tail fiber protein [Shigella phage Shfl2] (6e-122) {i}	0	N
255	157379-160687	+	1103	118.7	5.62	35	tail fiber protein [Shigella phage Shfl2] (2e-98) {i}	0	N
256	160719-161498	+	260	26.2	8.48	41	tail fiber protein [Shigella phage Shfl2] (5e-16) {i}	0	N
257	161529-162185	+	219	25.2	7.70	99	holin [Shigella phage Shfl2] (8e-158) {v}	1	N
258	162186-162458	-	91	10.7	5.42	53	anti-sigma factor [Shigella phage SP18] (1e-18) {iii}	0	N
259	162471-162623	-	51	6.0	4.92	98	hypothetical protein [Shigella phage Shfl2] (3e-25)	0	N
260	162620-162898	-	93	10.9	4.48	97	nuclease [Enterobacteria phage T4] (7e-56) {ii}	0	N
261	162888-163007	-	40	4.7	10.20	97	hypothetical protein [Shigella phage Shfl2] (9e-17)	0	N
262	162982-163200	-	73	8.8	8.51	98	hypothetical protein [Enterobacteria phage T4] (2e-22)	0	N
263	163184-163480	-	99	11.4	8.93	95	hypothetical protein [Shigella phage Shfl2] (4e-62)	0	N
264	163480-163941	-	154	18.0	5.15	99	hypothetical protein [Shigella phage Shfl2] (3e-109)	0	N
265	163938-164267	-	110	12.8	9.24	98	hypothetical protein [Shigella phage Shfl2] (6e-43)	0	N
266	164278-164913	-	212	23.7	7.78	76	hypothetical protein [Shigella phage SP18] (2e-56)	0	N

[a] i, phage structure and packaging; ii, DNA replication and modification; iii, signal transduction and regulation; iv, nucleotide metabolism; v, host lysis.
